# Risk Prediction for Early Mortality in Patients with Newly Diagnosed Primary CNS Lymphoma

**DOI:** 10.7150/jca.32467

**Published:** 2019-07-05

**Authors:** Chia-Hsin Lin, Ching-Fen Yang, Huai-Che Yang, Li-Yu Fay, Chiu-Mei Yeh, Ai-Seon Kuan, Hao-Yuan Wang, Jyh-Pyng Gau, Liang-Tsai Hsiao, Tzeon-Jye Chiou, Po-Min Chen, Yao-Chung Liu, Po-Shen Ko, Jin-Hwang Liu, Chia-Jen Liu

**Affiliations:** 1 Department of Radiation Oncology, Linkou Chang Gung Memorial Hospital Medical Center, Taoyuan City, Taiwan; 2 School of Medicine, National Yang-Ming University, Taipei, Taiwan; 3 Department of Pathology and Laboratory Medicine, Taipei Veterans General Hospital, Taipei, Taiwan; 4 Department of Neurosurgery, Taipei Veterans General Hospital; 5 Institute of Brain Science, National Yang-Ming University; 6 Division of Hematology and Oncology, Department of Medicine, Taipei Veterans General Hospital, Taipei, Taiwan; 7 Institute of Public Health, National Yang-Ming University, Taipei, Taiwan; 8 Cancer Epidemiology Unit, Nuffield Department of Population Health, University of Oxford, Oxford, OX3 7LF, UK; 9 Division of Transfusion Medicine, Taipei Veterans General Hospital, Taipei, Taiwan; 10 Institute of Biopharmaceutical Sciences, National Yang-Ming University, Taipei, Taiwan; 11 Chong Hin Loon Memorial Cancer and Biotherapy Research Center, National Yang-Ming University, Taipei, Taiwan

**Keywords:** Early mortality, epidemiology, primary CNS lymphoma, prognostic factors

## Abstract

**Background:** Overall survival of patients with primary CNS lymphoma (PCNSL) has improved since the introduction of immunochemotherapy. However, up to 10-15% of PCNSL patients still die shortly after diagnosis. In the present study, we aimed to investigate the risk factors of early mortality (death within 60 days after diagnosis) in patients with PCNSL.

**Methods:** We included newly diagnosed PCNSL patients in a tertiary medical center in Taiwan between January 1, 2002 and May 31, 2018. Clinical risk factors were collected and compared between PCNSL patients who had and did not have early mortality.

**Results:** A total of 133 consecutive patients with PCNSL were included in this study. Approximately 9.8% of the PCNSL patients had early mortality. In multivariate analysis, age ≥ 80 (adjusted hazard ratio [HR] 3.34, 95% confidence interval [CI] 1.01-11.04, *p* = 0.048) and involvement of the basal ganglia (adjusted HR 4.85, 95% CI 1.47-15.95, *p* = 0.009) were identified as independent risk factors of early mortality. Use of MTX-based chemotherapy served as an independent protective factor for early mortality (adjusted HR 0.19, 95% CI 0.05-0.67, *p* = 0.010). Infection and tumor-associated mass effect contributed most to early mortality.

**Conclusion:** Early mortality is not uncommon in patients with PCNSL. Identification of patients with higher risk may help clinicians with initiating appropriate surveillance and management.

## Introduction

Primary central nervous system (CNS) lymphoma (PCNSL) is a relative rare disease, accounting for approximately 4% of all primary CNS tumors 1-3. It is an extranodal variant of non-Hodgkin's lymphoma (NHL), which is confined to the brain, leptomeninges, spinal cord and eyes, and it is without systemic involvement 4, 5. Recently, a steady increase in incidence of PCNSL was reported, especially in elderly patients (≥ 65 years), which represents the majority of immunocompetent PCNSL 6, 7.

When compared with systemic diffuse large B-cell lymphoma (DLBCL), the prognosis of PCNSL is poor. PCNSL patients who do receive no treatment have a rapidly fatal course, with a median survival of 3.3 months 8. The prognosis of PCNSL patients, however, has substantially improved in the recent two decades due to the introduction of induction immunochemotherapy (rituximab, methotrexate, etc.) in combination with autologous hematopoietic stem cell transplantation (auto-HSCT) as consolidation for eligible patients 3, 9. Omuro *et al.* reported remarkable results in a phase-2 study for patients of newly diagnosed PCNSL with a three-year overall survival (OS) rate and progression-free survival (PFS) of up to 81% and 79%, respectively 10. Nevertheless, despite the advancement of novel therapies and improvement of OS, some patients still saw early mortality 10. Furthermore, patients with significant comorbidities or critical conditions were usually excluded from clinical trials, making the results not generalizable to all PCNSL patients 11-14.

To date, the International Extranodal Lymphoma Study Group (IELSG) score, Memorial Sloan Kettering Cancer Center (MSKCC) score, and the Nottingham-Barcelona (NB) model make up three major prognostic scoring systems 15, 16. Some parameters—for example, age, performance status (PS), serum lactate dehydrogenase (LDH) level, cerebrospinal fluid (CSF) total protein concentration, tumor localization (deep vs. superficial) and number of lesions—are well-established prognostic factors in the aforementioned models 15-17. However, the current scoring systems for PCNSL are used for prediction of OS. Hervé *et al*. further demonstrated that the prognostic values of some of these parameters may vary from time to time 18. Given that PCNSL is a heterogeneous disease, and the survival duration ranges from a month to more than five years,^15^, ^16^ the present scoring systems may not extrapolate to accurately predict the outcome of PCNSL patients during a particular period after diagnosis.^18^

However, few studies have delineated the incidence or risk factors related specifically to early mortality in patients with PCNSL. In addition, patients who had early mortality were not studied in randomized control trials, making the evidence from observational studies very important. Accordingly, we conducted a longitudinal observational study to address this issue.

## Patients and Methods

### Study Population

This study consecutively enrolled patients who were newly diagnosed with PCNSL between January 1, 2002 and May 31, 2018 at Taipei Veterans General Hospital. Patients were eligible if they had non-Hodgkin's lymphoma exclusively involving the CNS, leptomeninges, cranial nerves, or eyes. Diagnosis was made on stereotactic or surgical biopsy, CSF cytology analysis, or vitreous biopsy and was reviewed by at least one hematologist and one pathologist. Patients who had suspected PCNSL lesions demonstrated only by brain magnetic resonance image (MRI) or computed tomography (CT) but without histological confirmation were not included. Exclusion criteria were human immunodeficiency virus seropositivity, other immunodeficiency diseases or evidence of systemic NHL, as revealed by CT images of the chest, abdomen, pelvis and bone marrow aspiration and biopsy. Follow-up was continued until death, dropout, 60 days, or October 10, 2018. The Institutional Review Board of Taipei Veterans General Hospital issued a formal written waiver for the need for consent (no. 2016-05-003BC).

### Data Collection

Medical records for all PCNSL patients were reviewed. Clinical characteristics and laboratory data at diagnosis, such as age, sex, date of diagnosis, date of death, cause of death, comorbidities, treatment, chemotherapy regimen, LDH levels 15, hemoglobin levels, white blood cell (WBC) count, platelet count and albumin level were retrieved. Patients' serum β2-microglobulin level was measured by immunoassay (upper limit of normal, 1.61 mg/l). Cutoff values of hemoglobin, albumin, WBC counts, and platelet counts were 8.5 g/dl, 3 g/dl, 10,000/μl, and 100,000/μl, respectively, which are correlated with the early mortality seen in systemic NHL in previous studies 19, 20. Performance status is defined according to Eastern Cooperative Oncology Group (ECOG) criteria 15, 21, 22. There is no formal definition of early mortality; for the present study, we chose 60 days from disease diagnosis, according to studies of other hematologic malignancies 23, 24.

### Statistical Analysis

Patients' demographic and clinical characteristics with and without early mortality are presented as the total number (*n*) and proportion (%). Data are presented as medians and interquartile ranges (IQR) for skewed data. In the survival analysis, hazard ratios (HRs) and the 95% confidence intervals (CIs) were calculated using Cox proportional hazards models. We used multivariate analysis to calculate adjusted HRs while adjusting for possible confounding factors, including the demographic and clinical characteristics of patients. All risk factors with *p* < 0.1 in the univariate model were selected for stepwise-selection Cox proportional hazards model. To deal with immortal time bias, we analyzed treatment as time-dependent covariates in the Cox proportional hazards model. Treatments included radiotherapy and chemotherapy agents, such as methotrexate (MTX), vincristine, cyclophosphamide, cytarabine, rituximab, and corticosteroid. Data management and all statistical analysis was performed using SAS 9.4 software (SAS Institute Inc., Cary, NC). All statistically significant levels were set at *p* < 0.05.

## Results

### Clinical Characteristics of the Study Population

**Figure 1:**
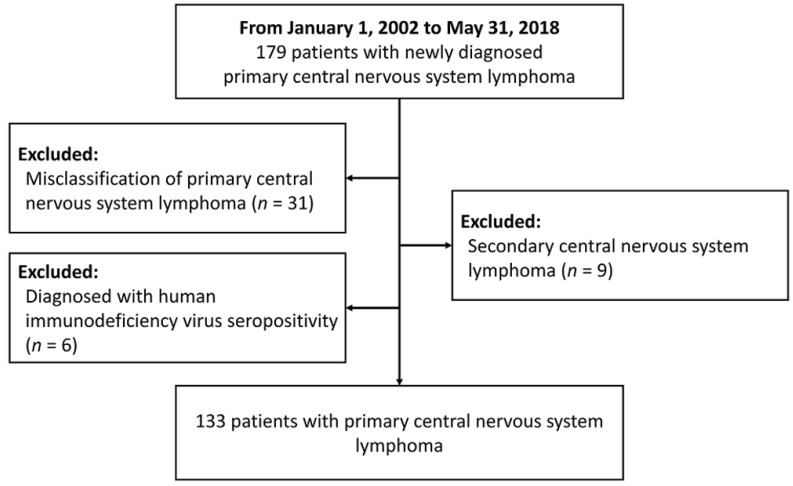
The flow chart for this study

**Figure 2:**
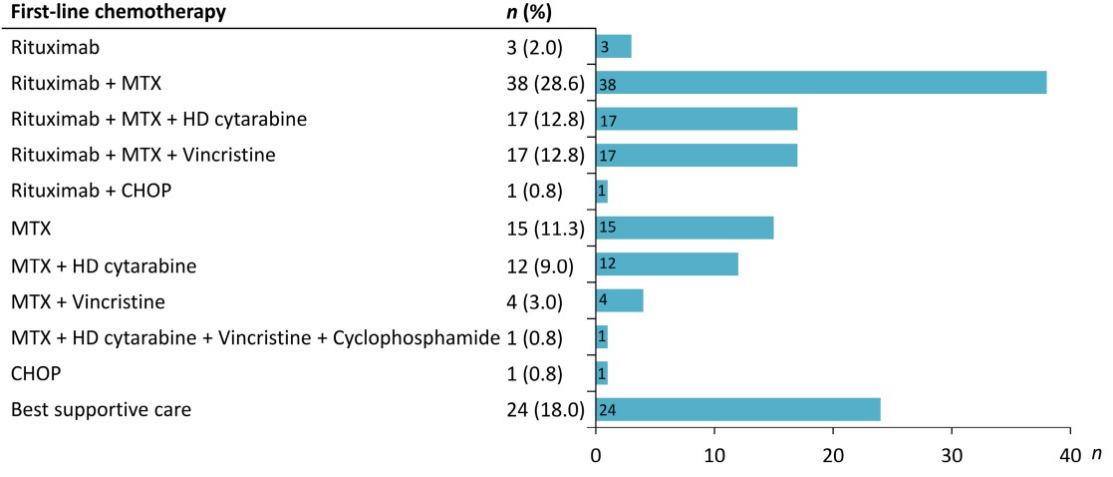
** Treatment in the 133 primary CNS lymphoma patients studied.** MTX, methotrexate; HD, high dose; CHOP, cyclophosphamide, doxorubine, vincristine, prednisone.

A total of 179 patients with CNS lymphoma diagnosed at Taipei Veterans General Hospital between January 1, 2002 and May 31, 2018 were identified. After reviewing the pathology and clinical data, patients who were misclassified into PCNSL (*n* = 31), diagnosed with secondary CNS lymphoma (*n* = 9) or with HIV infection (*n* = 6) were excluded. Finally, 133 PCNSL patients were enrolled in the study (Figure 1). Table 1 summarizes the clinical characteristics of PCNSL patients. The median age was 64 years (range 22-88 years), and 56.4% were male. Sixty-eight patients (51.1%) had an ECOG PS more than 1. The most common sites were frontal (38.4%) and basal (36.8%) lobe. The pathological diagnosis of all patients was diffuse large B-cell lymphoma. The details of treatment regimens are shown in Figure 2. Of the 133 PCNSL patients, 118 (88.7%) received treatment after initial diagnosis, and MTX was the most commonly used drug (*n* = 104) in the frontline treatment, followed by rituximab (*n* = 76), high-dose cytarabine (*n* = 30), and vincristine (*n* = 22). Radiotherapy was administered in 67 patients (50.4%). Radiotherapy was administered to the whole brain with a median dose (IQR) of 30 (24-40) Gy, followed or not by a tumor-bed boost with 41 (36-46) Gy.

### The Risk Factors of Early Mortality

In the univariate analysis, we found that old age, PCNSL with basal ganglia involvement, worse ECOG performance status, receiving MTX- and rituximab-based chemotherapy were significant predictors for early mortality in patients with PCNSL (Table 2). In the multivariate analysis, age ≥ 80 (adjusted HR 3.34, 95% CI 1.01-11.04, *p* = 0.048) and PCNSL with basal ganglia involvement (adjusted HR 4.85, 95% CI 1.47-15.95, *p* = 0.009) remained statistically significant. Meanwhile, frontline MTX lowered the risk of early mortality (adjusted HR 0.19, 95% CI 0.05-0.67, *p* = 0.01) after adjustment with potential confounding factors. Whole brain radiation therapy (WBRT) and surgical partial resection, were not associated with early mortality in either univariate or multivariate analysis.

### Incidence and Causes of Early Mortality

**Figure 3:**
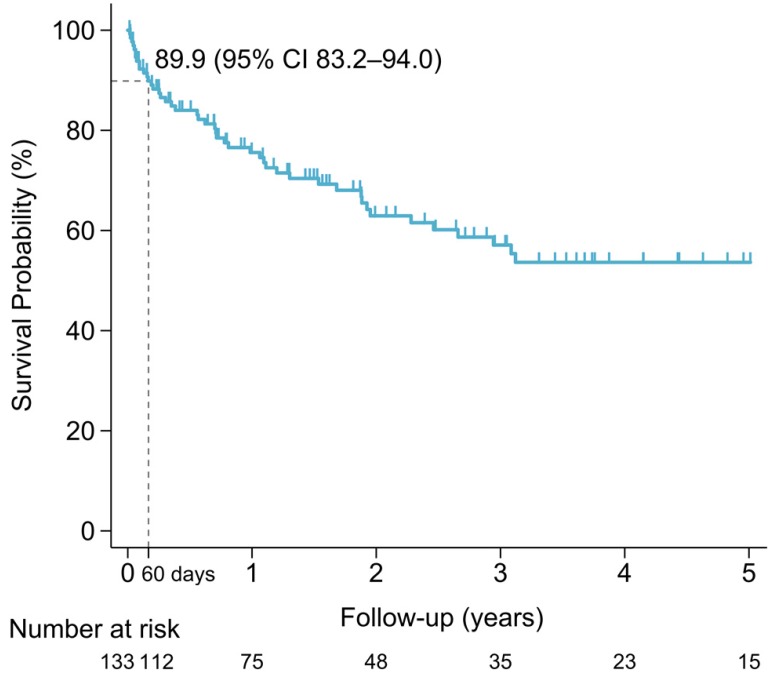
Survival probability of PCNSL patients

**Figure 4:**
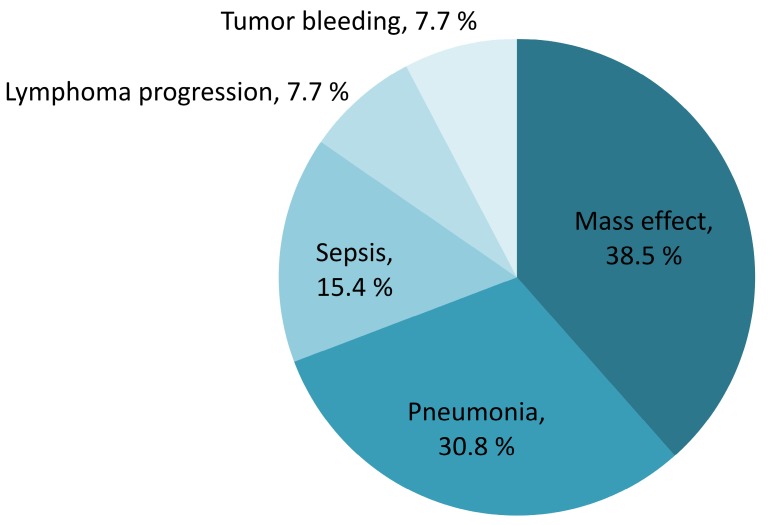
Contributing causes of early mortality (percentage of all mortality)

The median overall survival of the entire population was 8.4 years (IQR 2.5-not reach years). The sixty-day survival rate for PCNSL patients was 89.9% (95% CI 83.2%-94.0%). Early mortality arose in 13 of the 133 (9.8%) newly diagnosed PCNSL patients. The survival probability in PCNSL patients is shown in Figure 3. Mortality was related to PCNSL with mass effect in 5 (38.5%), tumor bleeding in 1 (7.7%) and disease progression in 1 (7.7%) of early-mortality patients. Infections were also direct causes of mortality in 46.2% of early-mortality PCNSL patients (*n* = 6). Pneumonia directly caused death in four cases and sepsis attributed to two cases, accounting for 66.7% and 33.3% of the patients who died early of infection, respectively. The causes of early mortality are shown in Figure 4.

## Discussion

Knowledge of risk factors and causes of deaths in PCNSL patients may help clinicians with identifying higher-risk patients and developing strategies for prevention of early mortality. However, clinical factors that affect early mortality have scarcely been studied and so far less investigated in clinical trials. To the best of our understanding, this is the largest study that examines risk factors of early mortality in PCNSL patients.

Our study reveals that old age (age ≥ 80) and PCNSL with basal ganglia involvement were potential risk factors of early mortality (death within 60 days) in PCNSL patients. Our results are consistent with studies that reported factors predicting overall survival at a later stage 15, 25, 26. This is also the first study that reports an association between MTX-based chemotherapy and reduced risk of early mortality in PCNSL patients. Furthermore, we report that infection and tumor-related mass effect are two major causes of early mortality among PCNSL patients.

Age serves as an independent survival predictor due to its association with comorbidity and as an indicator for the possibility of toxic death attributed to chemoradiation 27, 28. Two large retrospective studies, namely IELSG and MSKCC, showed age as an independent parameter for survival and combined age with other factors to establish models for PCNSL discriminating between different prognostic groups 15, 16. Recent studies found that age may confer a time-dependent impact and might play a role in predicting early death. Herve *et al.* retrospectively evaluated 91 patients with PCNSL, finding that age impacted survival during the initial period but lost its prognostic value after six months 18, which was the approximate time of completion of PCNSL treatment. Our study results are consistent with previous studies, showing age as an independent risk factor for survival, and further identified an age cutoff at 80 conferring clinical relevance for early mortality within 60 days.

This study indicates that involvement of the basal ganglia is an independent predictor for early mortality in PCNSL patients. The basal ganglia consist of multiple subcortical regions intertwined with the cerebral cortex, thalamus and brain stem and serves a pivotal role in movement adjustment and initiation. Previously, deep brain involvement, which is defined in IELSG as “including involvement of periventricular regions, the basal ganglia, brainstem or cerebellum,” has long been widely reported as an unfavorable prognostic factor for death within six months and for overall survival 7, 15, 18. In this study, evidence indicated that PCNSL involving specific location such as basal ganglia rather than brain stem or cerebellum was associated with higher risk of early mortality within 60 days. Elucidation of the mechanism and external validation for the prognostic value of basal ganglia involvement are definitely required in the future prospective study.

Performance status (PS) has long been accepted as a powerful prognostic factor for PCNSL, which plausibly dictates the treatment plan of patients (doses and chemotherapy regimens). In databases from previous retrospective study (IELSG, MSKCC and Nottingham/Barcelona), PS consistently determined survival of PCNSL patients 15-17. In our cohort, ECOG more than 1 was associated with early mortality in univariate analysis. Nonetheless, ECOG PS was not a significant risk factor for early mortality in multivariate analysis. This may be explained by the strong correlation between PS and age, of which the effect outweighing that of PS on predicting early mortality. Also, ECOG PS scales have been long criticized for its subjective nature, thus leading to high interobserver variability and recall bias 29.

We further analyzed the potential impact of first-line therapies on early mortality and found that MTX was associated with a significantly reduced risk of early mortality within 60 days. Although no universal consensus has been reached regarding the optimal treatment for PCNSL, MTX-based chemotherapy is the primary building block for the combination of polychemotherapy currently. With the introduction of MTX, prolonged median OS of 30 to 60 months has been observed compared with WBRT alone 3, 30-33. This study corroborated previous data showing beneficial effect of MTX toward PCNSL patients and further reported MTX as an independent protective factor against early mortality in PCNSL patients.

Immunochemotherapy with rituximab was another potential first-line PCNSL treatment regimens 3, 34. In our study, individuals receiving immunochemotherapy with rituximab showed lower risk of early mortality in univariate analysis but did not attain statistical significance in multivariate analysis. As a monoclonal anti-CD20 antibody, rituximab has been shown to confer significant benefits in the treatment of systemic DLBCL.^35^ However, the large molecular weight of rituximab limits its penetration across the blood-brain barrier, thus rendering the role of rituximab a long-debated issue in the management of PCNSL.^36^, ^37^ A meta-analysis including six retrospective studies and two phase II randomized controlled trials (RCT) demonstrated that the addition of rituximab was significantly associated with higher complete remission rate, PFS and OS 38. Nevertheless, the interpretation should be cautious given that one of the included RCT was of poor quality and the heterogeneity among studies was significant with respect to two-year PFS. Additionally, the most recent phase III international RCT (*n* = 200) reported that the incorporation of rituximab into MTX-based regimens did not improve response rates or survival 39. Herein, we also did not detect significant benefit of rituximab in early mortality of PCSNL after adjustment for other confounding variables, suggesting a marginal role of rituximab regarding early mortality. Certainly, further validation of the contribution of rituximab to early mortality awaits more prospective studies.

CSF protein may reflect tumor burden, leptomeningeal involvement and even disease severity. In the large database form IESLG, CSF protein concentration independently determined survival of PCNSL patients 15. Yet, several studies later cast doubt on the prognostic value of CSF protein. D'Haene *et al.* exhibited that the IESLG score without this parameter remains potent in predicting survival independently.^40^ Similarly, in another study of 91 PCNSL patients, CSF protein level was not identified as a significant predictive factor in both univariate and multivariate analysis 18. The present study further demonstrated that increased CSF concentration was not associated with early mortality within 60 days for PCNSL patients. Both our results and previous studies suggest that CSF protein level may not have significant implications for predicting the prognosis of the disease. Surely, more prospective studies are warranted to examine the impact of CSF protein concentration on PCNSL survival prognostication. LDH serum level is a potential indicator of tissue breakdown and therefore may denote the proliferation rate of tumors. It has been considered an independent factor for predicting OS in PCSNL patients in IELSG 15. Herve *et al*. also established the role of serum LDH level in predicting PCNSL survival within six months 18. However, results from two large cohorts were inconsistent with the aforementioned findings, thereby raising questions in terms of the prognostic value of serum LDH level for survival 16, 41. In current study, heightened LDH serum level, was also not identified as a risk factor for early mortality within 60 days in either univariate or multivariate analysis. The controversy for the role of serum LDH in predicting OS and early mortality for PCNSL remains and thus requires further study.

In the current study, we reported that around 9.8% of PCNSL patients suffered from early mortality, with tumor-associated mass effect and infection as two leading causes. Those who succumb to early mortality lose the opportunity of receiving complete cycles of standardized chemotherapy regimens and further advanced therapies such as auto-HSCT. Ferreri *et al*. reported that more than 10% of patients encountering early mortality before finishing four 21-day cycles of a MTX-based regimen, largely due to acute toxic effect and lymphoma progression.^11^ In a phase-3 randomized trial (*n* = 526), Thiel *et al*. also recorded an estimated 66 patients (13.0%) having died during front-line chemotherapy (six 14-day cycles of MTX) before receiving whole brain radiation, majorly related to treatment and disease progression 12. Knowledge of the main causes and predicting factors for early mortality can help clinicians identify those at-risk patients, take special precautions and even develop modified chemotherapy regimens to extend their survival time. Prior study has investigated the predicting value of IELSG score for early mortality in the earlier cohort (1984-2006) 18. However, the treatment and survival outcomes for PCNSL patients have improved significantly over the past decades, as indicated by some specialized centers 10, 13, 14 as well as large national databases 42, 43. Therefore, the results by Ghesquières *et al*. may not be directly generalizable to patients in the contemporary era, thereby highlighting the importance of this research as we investigated patients in more recent periods (2002-2018). When a high-risk population can be identified, prophylactic measures such as prophylactic antimicrobials or approaches for early detection of infections could be prompted and further tested in prospective studies considering infection as a main cause of early mortality.

Our study includes some limitations. The retrospective nature of this study precludes us from drawing solid conclusions from these data. Second, this study spanned an observation period of 16 years, during which the standards of care and diagnosis undoubtedly varied. Furthermore, considering the very small cohort (*n* = 133), it is of difficulty to determine risk factors for early mortality, which could therefore reduce the chance of detecting a true effect. Yet, the extreme rare incidence of PCNSL itself limits the number of patients to be enrolled, making the evidence from the present observational study very important given it is the largest cohort addressing the issue of early mortality of PCNSL to date. Of note, this study may not represent all early-mortality PCNSL patients given that those with suspected PCNSL lesions only in images but without histological confirmation were not included. However, the strict criteria ensure definite diagnosis and prevents the misclassification of patients without PCNSL into this cohort. Finally, the timing of initiating cancer treatment and the choice of which chemotherapy regimens selected as first-line therapy are physician-dependent rather than a standardization of care, and thus it is difficult to evaluate the impact of treatment on early mortality without introducing some unknown confounding factors or bias.

## Conclusion

Despite the development of innovative combination of immunochemotherapies, treating PCNSL remains a challenge. This cohort showed that early mortality occurred in nearly 9.8% of newly diagnosed PCNSL patients and that infection and tumor-associated mass effect were two leading causes of mortality in these patients. We further identified the risk factors of early mortality, including age ≥ 80 and basal ganglia involvement. Additionally, individuals receiving MTX-based chemotherapy showed decreased risk of early mortality. Identifying risk factors for early mortality might assist physicians in identification of these patients, as well as the initiation of intensive surveillance and development of prophylactic measures. However, more evidence from prospective studies is needed to further confirm these risk factors.

## Figures and Tables

**Table 1:** Baseline PCNSL patient characteristics

Characteristics	Total*	Early mortality (60 days)	Non-early mortality
	*n* = 133	*n* = 13	*n* = 120
	*n* (%)	*n* (%)	*n* (%)
Median age, years (range)	64 (22-88)	70 (44-87)	64 (22-88)
≥ 80	16 (12.0)	4 (30.8)	12 (10.0)
< 80	117 (88.0)	9 (69.2)	108 (90.0)
Sex			
Male	75 (56.4)	8 (61.5)	67 (55.8)
Female	58 (43.6)	5 (38.5)	53 (44.2)
Site			
Frontal lobe	51 (38.4)	5 (38.5)	46 (38.3)
Parietal lobe	37 (27.8)	3 (23.1)	34 (28.3)
Temporal lobe	37 (27.8)	4 (30.8)	45 (37.5)
Occipital lobe	23 (17.3)	4 (30.8)	21 (17.5)
Basal ganglia	49 (36.8)	9 (69.2)	40 (33.3)
Brain stem	17 (12.8)	2 (15.4)	15 (12.5)
Cerebellum	18 (13.5)	3 (23.1)	15 (12.5)
Meninges	1 (0.8)	0 (0.0)	15 (12.5)
Cranial nerves	2 (1.5)	0 (0.0)	2 (1.7)
Median max diameter (IQR)	3.4 (2.6-4.5)	4.0 (3.2-4.3)	3.3 (2.6-4.5)
Number of lesions			
1	66 (49.6)	8 (61.5)	58 (48.3)
2	27 (20.3)	2 (15.4)	25 (20.8)
3	17 (12.8)	0 (0.0)	17 (14.2)
4	8 (6.0)	0 (0.0)	8 (6.7)
≥ 5	15 (11.3)	3 (23.1)	12 (10.0)
Deep brain lesions	91 (68.4)	11 (84.6)	80 (66.7)
Midline shift	46/131 (35.1)	6/13 (46.2)	40/118 (33.9)
Initial surgical management			
Stereotactic biopsy	85 (63.9)	9 (69.2)	76 (63.3)
Open biopsy	6 (4.5)	0 (0.0)	6 (5.0)
Partial resection	42 (31.6)	4 (30.8)	38 (31.7)
CSF involvement	5/74 (6.8)	0/2 (0.0)	5/72 (6.9)
Intraocular involvement	15/63 (23.8)	0/4 (0.0)	15/59 (25.4)
ECOG			
0-1	65 (48.9)	2 (15.4)	63 (52.5)
≥ 2	68 (51.1)	11 (84.6)	57 (47.5)
Lab data, median (IQR)			
White blood cell, /ul	9,700 (7,200-13,200)	10,400 (6,700-13,600)	9,650 (7,200-13,150)
Hemoglobin, g/dl	12.7 (11.4-14.1)	12.0 (10.6-12.7)	12.9 (11.7-14.1)
Platelet, /ul	199,000 (157,000-270,000)	171,000 (143,000-288,000)	200,500 (158,000-268,500)
Albumin, g/dl	3.7 (3.2-4.1)	3.5 (3.0-3.8)	3.8 (3.3-4.1)
Lactate dehydrogenase, U/L	241.0 (198.0-317.0)	265.0 (235.0-501.0)	236.5 (194.5-312.5)
CSF protein, mg/dl	66.9 (43.1-100.0)	78.6 (74.3-241.1)	63.5 (43.0-100.0)
First-line treatments			
MTX	104 (78.2)	4 (30.8)	100 (83.3)
Rituximab	76 (57.1)	2 (15.4)	74 (61.7)
Vincristine	22 (16.5)	0 (0.0)	22 (18.3)
Cyclophosphamide	1 (0.8)	0 (0.0)	1 (0.8)
Cytarabine	30 (22.6)	0 (0.0)	30 (25.0)
Corticosteroid	127 (95.5)	10 (76.9)	117 (97.5)
Radiotherapy	67 (50.4)	2 (15.4)	65 (54.2)

IQR, interquartile range; BM, bone marrow; ECOG, Eastern Cooperative Oncology Group performance; MTX, methotrexate. * Including some missing value

**Table 2:** Analysis of risk factors for mortality (60 days) in patients with PCNSL

Predictive variables	Univariate analysis			Multivariate analysis^a^	
	HR (95% CI)	*P* value		HR (95% CI)	*P* value
Age ≥ 80	3.72 (1.14-12.10)	0.029		3.34 (1.01-11.04)	0.048
Sex (male)	1.30 (0.43-3.98)	0.642			
Site					
Frontal lobe	0.94 (0.31-2.88)	0.914			
Parietal lobe	0.78 (0.22-2.84)	0.709			
Temporal lobe	0.74 (0.23-2.40)	0.614			
Occipital lobe	0.93 (0.21-4.20)	0.926			
Basal ganglia	4.26 (1.31-13.85)	0.016		4.85 (1.47-15.95)	0.009
Brain stem	1.36 (0.30-6.14)	0.688			
Cerebellum	2.05 (0.57-7.47)	0.274			
Meninges	*	0.995			
Cranial nerves	*	0.993			
Max diameter ≥ 3.4	2.18 (0.67-7.08)	0.195			
Number of lesions ≥ 5	2.58 (0.71-9.37)	0.150			
Deep brain lesions	2.70 (0.60-12.20)	0.196			
Midline shift	1.62 (0.54-4.82)	0.385			
CSF involvement	*	0.998			
Intraocular involvement	*	0.996			
ECOG ≥ 2	5.48 (1.21-24.70)	0.027			
Laboratory data					
CSF protein ≥ 66.9 mg/dl	*	0.995			
WBC ≥ 10,000/ul	1.32 (0.44-3.94)	0.615			
Hemoglobin < 8.5 g/dl	*	0.994			
Platelets ≥ 100,000/ul	*	0.993			
ANC ≥ 7,278/ul	1.12 (0.38-3.35)	0.833			
Albumin < 3 g/dl	1.85 (0.40-8.59)	0.430			
Lactate dehydrogenase ≥ 250 U/L	1.57 (0.48-5.16)	0.454			
Serum β2-microglobulin ≥ 1,613 mg/l	1.83 (0.17-20.16)	0.622			
Chemotherapy^b^					
MTX	0.20 (0.06-0.70)	0.012		0.19 (0.05-0.67)	0.010
Rituximab	0.19 (0.04-0.91)	0.037			
Radiotherapy	1.65 (0.35-7.70)	0.524			

HR, hazard ratio; CI, confidence interval; ECOG, Eastern Cooperative Oncology Group performance. ^a^All factors with *p* < 0.1 in the univariate analysis were selected for stepwise-selection Cox proportional hazards model. ^b^Treatment was analyzed as a time-dependent covariate in the Cox regression model.

## Abbreviations

CNS: central nervous system
PCNSL: primary CNS lymphoma
NHL: non-Hodgkin's lymphoma
DLBCL: diffuse large B-cell lymphoma
auto-HSCT: autologous hematopoietic stem cell transplantation
OS: overall survival
PFS: progression-free survival
IELSG: International Extranodal Lymphoma Study Group
MSKCC: Memorial Sloan Kettering Cancer Center
NB: Nottingham-Barcelona
PS: performance status
LDH: lactate dehydrogenase
CSF: cerebrospinal fluid
MRI: magnetic resonance image
CT: computed tomography
WBC: white blood cell
ECOG: the Eastern Cooperative Oncology Group performance score
IQR: interquartile ranges
HRs: hazard ratios
CI: confidence interval
MTX: Methotrexate
WBRT: whole-brain radiation therapy
